# Monthly Summary

**Published:** 1876-03

**Authors:** 


### MONTHLY SUMMARY.

*Subcutaneous Injection of Water.*—Dr. LELUT, in a communication to *L' Union Medicale* of October 5th, states that, during the last three months, he has employed subcutaneous injection of water only for the relief of pain, with the most successful results. He relates how he was induced to adopt this method of treatment by pure accident. He had left a bottle containing a solution of morphia on his desk. His servant upset the bottle, and filled it with water to conceal her carelessness. The next day Dr. Lelut, having occasion to repeat a hypodermic injection in the case of a patient suffering from sciatica, used the liquid from the usual bottle. When he saw his patient the next day, he found him in a most lively frame of mind, and was thanked by him in the following terms: "Oh! doctor, how grateful I am to you. You relieved all my pain without making me feel sick." He was astonished at this result in a patient who had suffered from nausea and vomiting after each injection. He naturally proceeded to examine the solution used, and was astonished to find only pure water in the bottle. He repeated the experiment during the ensuing days on several patients, and invariably found that he gave them relief and avoided nausea and vomiting; he has consequently, as above stated, continued to employ the same plan from June last up to the present time, with, as a rule, satisfactory results.—*Brit. Med. Journ.*

---

*Chloride of Lead as a Deodorizer and Disinfectant.*—Dr. R. H. Goolden calls attention to the value of chloride of lead, which he says is the most powerful and economical deodorizer and disinfectant.

To prepare it for use he directs to take half a drachm of nitrate of lead, dissolve it in a pint or more of boiling water, then dissolve two drachms of common salt in a bucket of water, and pour the two solutions together; allow the sediment to subside. The clear supernatent fluid will be a saturated solution of chloride of lead. A cloth dipped in this solution and hung up in a room sweetens a fetid atmosphere instantaneously, or the solution thrown down a sink, water-closet, or drain, or over a heap of dung or other refuse, will produce a like result. Even the tarnishing of gold and silver plate may be prevented by a rag dipped in the solution being hung up in the room or window where it is exposed.

He relates some striking instances of the instantaneous and efficient action of this preparation.—*London Lancet.*

*Deaths from Chloroform.*—A recent death from chloroform in the practice of Mr. Chesshire, one of the surgeons of the Birmingham Eye Hospital, presents some peculiar features. The patient was a gardener, forty-two years of age, who had received a gunshot wound in his right eye while beating for game on the 16th ultimo. The safety of the other eye being endangered, Mr. Chesshire recommended extirpation of the affected eye-ball. Forty-five drops of chloroform were administered on a towel, and the patient "immediately became semi-unconscious." The operation was commenced, but, the patient not appearing to be sufficiently under the influence of chloroform, forty or fifty drops more were given. Mr. W. M. Jackson, of Smethwick, noticing a failure in the patient's pulse, at once seized him by the feet and lowered his head. He afterwards practised artificial respiration, but the man died in about half an hour from the time of the first administration of the chloroform. The patient had no disease of the heart, and was free from excitement prior to the operation. It is remarkable how small a quantity of chloroform proved fatal in this case; probably not more than two drachms were used altogether, and every precaution was taken in the administration of the drug, and yet death resulted.—*Lancet*, Dec., 11, 1875.

"We have heard of another death recently in London," it is stated in the *Brit. Med. Journ.* (Dec. 11,) "in the practice of a metropolitan hospital surgeon, and of which we have seen as yet no public notice.—*Med. Newc & Library.*

---

*Dislocating the Jaw for Chloroform Narcosis.*—Dr. Fleiberg, of Christiania, recommends the surgeon to deliberately dislocate the jaw, instead of pulling the tongue out with forceps, &c. It is easy to do this by placing both thumbs behind the symphysis, and both index fingers on the posterior edges of the rami, and dragging the bone forward He seems to do it as a preventive, for he speaks of having done it *a thousand times.* Langenbeck tells us that he and Esmarch have often done this. Perhaps these surgeons will tell us if dislocation often occurs spontaneously to those who have thus been treated.—*The Doctor.*

---

*An Excellent Tooth-wash.*—An excellent tooth-wash, very much resembling *Sozodont*, is made as follows:

| | |
|---|---|
| Carbonate of Potassa, - - - | ½ ounce. |
| Honey, - - - - - | 4 ounces. |
| Alcohol, - - - - - - | 2 " |
| Water, - - - - - - | 10 " |
| Oil Wintergreen, } Oil Rose, } - - - - | q. s. |

—*Druggists Advertiser.*

*Bi-carbonate of Soda as a Toothache Remedy.*—Dr. Duckworth, of St. Bartholomew's Hospital, London, has recently successfully used Bicarbonate of soda as a remedy for toothache, when applications of chloroform, either externally to the cheek or to the ear, or placed on cotton in the decayed tooth, failed ; and when carbolic acid applied as last mentioned also proved inoperative. Pledgets of cotton soaked in a solution of 30 grains of Bicarbonate of Soda dissolved in a fluid ounce of water gave instant relief. Dr. Duckworth considers that very frequently the pain is due to the contact of acid saliva with the decayed tooth, and therefore it is important in cases of odontalgia, first to determine whether the saliva has an acid reaction. If this be the case, then a simple alkaline application, as above stated, is the most efficacious means of cure.—*Druggists Advertiser.*

---

*Cancer of Tongue and Jaw.*—Dr. Post presented several specimens, the first of which was one of malignant growth of the floor of the mouth, the lower surface of the tongue, and the parts in front, involving the bone itself, and the integument in front of it. The latter condition was not recognized, however, until the time of the operation. The patient, a male, æt. 50 years, was admitted to the Presbyterian Hospital three months since, presenting an indurated ulcer below the tongue, concerning the diagnosis of which between cancer and syphilis there was at first some difficulty. Anti-syphilitic remedies were employed, but with no marked effect, and the patient's condition after a time grew gradually worse, being unable to swallow except with great difficulty, and suffering a great deal of pain. The suspicion of the malignancy of the growth being confirmed, and the prospects of the patient being desperate, an operation was determined upon, for the purpose of a temporary relief to his sufferings. It was performed by dividing the lower lip from each angle of the mouth downwards, and making a section of the jaw at the corresponding points. The tongue was drawn forward, a large portion of which was removed. It was then found that the morbid growth had perforated the jaw and involved the integument in front. A considerable but not an excessive quantity of blood was lost in the operation.

On the morning after the operation the patient was able to swallow without much difficulty. His pulse was fair, but he was quite restless, which latter was in a measure accounted for by the absence of a large portion of his tongue and the drawing forward and fixing of the remaining portion to guard against the danger of falling into the larynx. The patient died in less than twenty-four hours after the operation, from nervous prostration, which probably was somewhat connected with the habit of opium-eating which he had acquired.—*N. Y. Pathological Society.—Med. Record.*

*Collodion in Treatment of Carious Teeth.*—Dr. Lardier advises, in *L' Union Medicale,* the use of collodion in the treatment of carious teeth, by dropping the collodion into the cavity, after the latter has been cleaned and dried. The ether evaporates and narcotizes the nervous twigs. By solidifying, the collodion protects the tooth from the contact of the air.

Dr. Lardier states that he has thus succeeded in relieving many patients.

---

*Where to Look for Arsenic in Cases of Poisoning.*—Observa tions of great toxical importance have lately been made by Scoloscuboff (*Soc Ch.* August, 1875) with reference to the distribution of arsenic in the tissues of animals in cases of poisoning. Contrary to general belief, he finds the arsenic specially condensed in the nervous tissues. The experiments were made with dogs, rabbits and frogs. Dogs bear large doses of arsenic readily, taking without difficulty fifteen to eighteen times the quantity which, weight for weight, would be fatal to man. A bull dog took for thirty-four days gradually increasing quantities of sodium arsenite in his food, rising from 0.075 grains to 2.2 grains a day. The results of the poisoning were acute and quite marked. Calling the amount of arsenic found in 0.22 lb. of muscle 1, that in the same weight of liver was 10.8; brain, 36.5; spinal cord, 37.3. A dog of 24 1-5 lbs. weight was killed by subcutanious injection of sodium arsenite in 17 hours. The arsenic from the brain gave a decided reaction, that from the spinal cord was less, while in the liver and muscles only traces could be detected.—*Scientific American.*

---

*Warts.*—Dr. Guttceit, in the *St. Louis Clinical Record,* recommends rubbing warts, night and morning, with a moistened piece of muriate of amimonia. They soften and dwindle away, leaving no such white mark as follows their dispersion with lunar caustic.

---

*Death from Inhalation of Ether.*—At the New York Pathological Society, a case of death was reported as having occurred in the Homœopathic Hospital, from the inhalation of ether. The patient had necrosis of the jaw, and an operation was determined on. He was etherized and an incision made. At this stage he became cyanotic, and was held up by the heels and otherwise treated, but death supervened in ten minutes from the commencement of the inhalation. The quantity of ether consumed was two and a quarter ounces. Dissection revealed a fatty heart but no other lesion.—*Med. and Surg. Reporter.*

*Medical Diploma for Women*—The *British Medical Journal* states that the Council of the Royal College of Surgeons of England has arrived at the important decision to admit women to examination for its license in midwifery. This diploma will entitle them to a place on the Medical Register, and will give them a legally recognized position as practiti:ners in the obstetric department of medicine and surgery. The clause in the college charter under which the right to admission has been claimed, was, it appears, expressly framed, by the use of the word "persons," so meet the case of female as well as of male practitioners, and the college has been advised that it oould not lhgallp refuse to admit duly educated women to examination for this diploma.—*Medical and Surgical Reporter.*

---

*Camphor Poisoning.*—While some camphor was being weighed out, a lad of thirteen picked up two small pieces and took them away with him, for the purpose of floating and burning them. Soon afterward he began nibbling the camphor, and, as it afterward appeared, liking the taste of it, continued to do so until he he had eaten the two pieces. This was about four o'clock in the afternoon. Four hours afterward the child was with his brother in the dispensary, looking on, and was observed to do something which elicited the remark, "Are you dreaming?" No reply was given by the child, and it was noticed that something was wrong with him: his eyes were fixed in a stare, and he stood motionless and unconscious, His brother took him up to carry him into an adjoining room, where his father was, when he immediately became convulsed and perfectly rigid, with his head and legs bent back, so that he could only be placed on his side upon the floor. The convulsions increased until the flesh from the head to the shoulders became purple, and the pulse decreased rapidly until it could not be felt. The body then lost its rigidity and was apparently lifeless, but in about ten seconds the pulse could again be felt, the convulsions returned, and the child foamed at the mouth. Applications of cold water brought him round in about four minutes; violent vomiting then ensued; the child was for a time hysterical, but within an hour from the first attack he was so far recovered that he could be put to bed. The child afterward described the pieces of camphor which he ate as being each half the size of his thumb, and the assistant, who noticed him take one piece, thought it must have been about sixty grains in weight. The effects produced in this case were more severe than in any of those previously reported, and there was the further differences that unconsciousness preceded the convulsions, while in the other cases it followed them.—*Pharmaceutical Journal.*

